# Egg Formation in Lepidoptera

**DOI:** 10.1673/031.009.5001

**Published:** 2009-07-09

**Authors:** William H. Telfer

**Affiliations:** Emeritus Professor of Biology, University of Pennsylvania

**Keywords:** ovariole, follicle, octet, nurse cells, oocyte, follicle cells, gap junctions, tight junctions, intercellular bridges, ring canals, fat body, endocytosis, pinosomes, hemolymph proteins, vitellogenin, lipophorin, microvitellogenin, trypan blue, ion pumps, vitellin membrane, chorion, edysteroids, juvenile hormone, cAMP, PKA, G-proteins

## Abstract

Reproductive biology in the Twentieth Century produced comprehensive descriptions of the mechanisms of egg formation in most of the major orders of insects. While many general principles of ovarian development and physiology emerged, every order turned out to have a set of its own special motifs. Discovery of the lepidopteran motifs is summarized in this essay. The emphasis is on developmental mechanisms, beginning with the early growth and differentiation of female germ cells and ending, after many turns in morphogenesis, physiology and biosynthesis, with eggs that are filled with yolk and encased in chorions. Examples of uniquely lepidopteran traits include the cellular composition of ovarian follicles, the number of tubular ovarioles in which they mature, the functions of cell-to-cell junctional complexes in their maturation, their use of glycosaminoglycans to maintain intercellular patency during vitellogenesis, the role of proton and calcium pumps in their ion physiology, a separate postvitellogenic period of water and inorganic ion uptake, and the fine structure and protein composition of their chorions. Discovery of this combination of idiosyncracies was based on advances in the general concepts and techniques of cell and molecular biology and on insights borrowed from studies on other insects. The lepidopteran ovary in turn has contributed much to the understanding of egg formation in insects generally.

## Follicles and ovarioles

**Figure 1.**
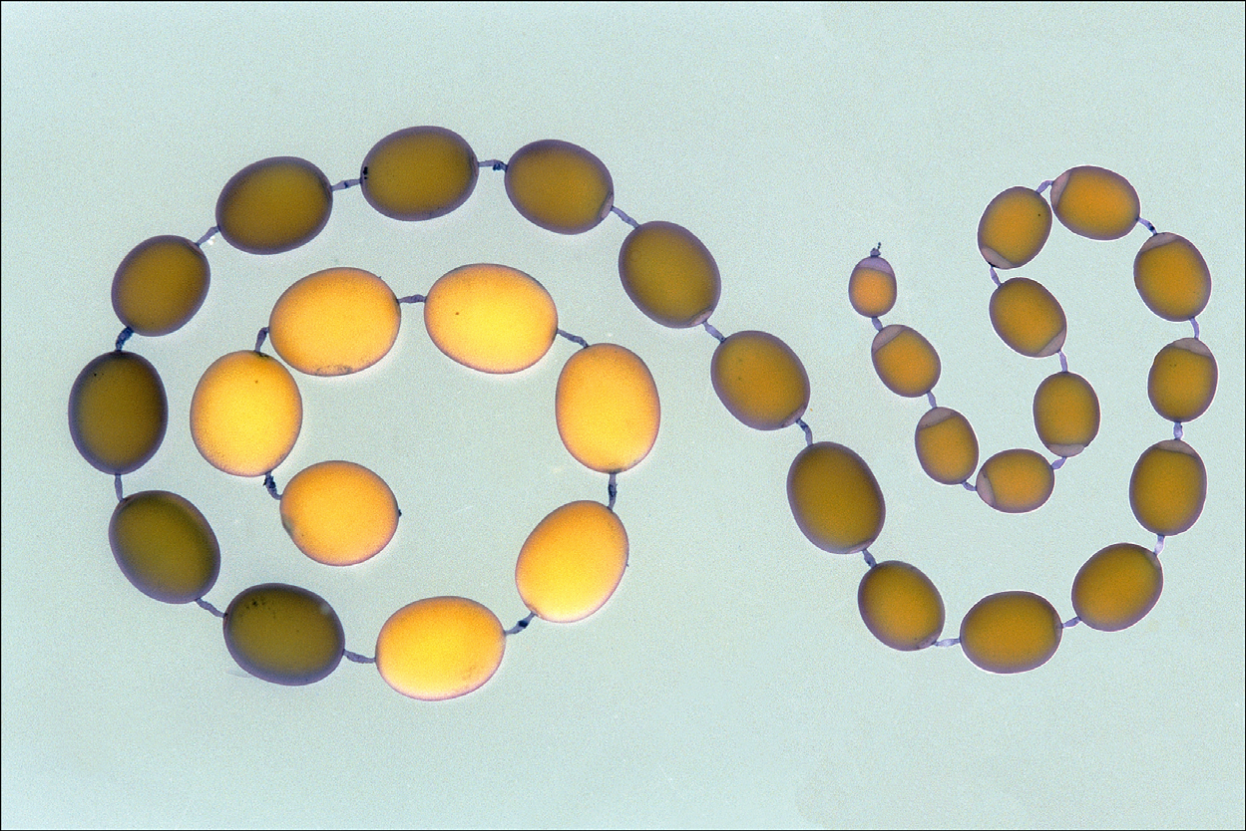
A chain of follicles dissected from an ovariole from a pharate adult of *Hyalophora cecropia*. About four hours of developmental time separate neighboring follicles. This chain had been stained *in vitro* for 5 min with trypan blue, which binds to vitellogenic follicles while leaving post-vitellogenic follicles unstained. The largest staining follicle is 2.0 mm long.

Every egg is the product of an ovarian follicle. In mature female lepidopterans the ovaries contain hundreds or thousands of these units arranged in eight tubular ovarioles. New follicles form periodically near the apex of each ovariole, and mature eggs are released into an oviduct at its base. Graded intermediate stages line up between these extremes (Figure 1), with the lag in developmental time between successive members related to the periodicity of follicle formation at the apex.

**Figure 2.**
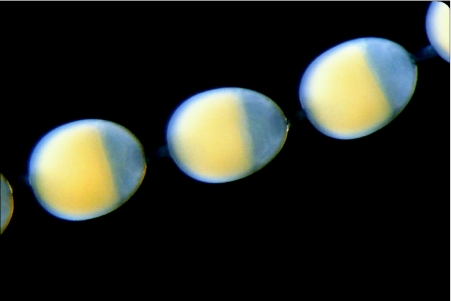
Three early vitellogenic follicle from an ovariole such as that in figure 1. Oocytes are opaque due to light scattering by yolk particles and yellow due to the carotenoid conjugates of lipophorin, the second most abundant yolk protein in this species. Nurse cells form a cap on the anterior surface of the oocyte. The translucent layer surrounding the oocyte is the 50 µm thick epithelium of follicle cells

**Figure 3.**
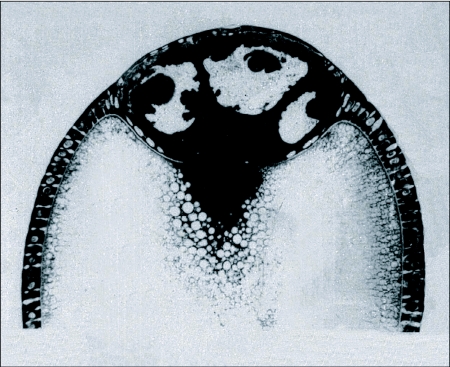
Section of a vitellogenic *Hyalophora* follicle that had been stained with Azure-B to reveal the distribution of RNA. Cytoplasm is deeply stained. Three giant, folded nurse cell nuclei are at the top of the section. A flattened epithelium of follicle cells covers the nurse cells while the oocyte is covered by taller, columnar-shaped cells. The oocyte is close-packed with non-staining yolk particles. An RNA-rich delta of cytoplasm lies close to the nurse cells at its anterior pole and a lightly staining cortex underlies its surface (Reprinted with permission from Pollack and Telfer 1969 *Journal of Experimental Zoology* 190:1–24.)

Without known exception, each lepidopteran follicle includes at the outset a single oocyte and seven nurse cells encased in an epithelium of follicle cells (Figures 2, 3). There is a clear division of labor among these components. The nurse cells are mitotic siblings of the oocyte and attach to it and to each other by open bridges of cytoplasm. The chromosomes of the nurse cells undergo endopolyploid replications that generate DNA levels as high as 64,000 genome copies per nucleus (Cardoen et al. 1990). They generate cytoplasm for the egg. The oocyte, by contrast, remains in first meiotic prophase throughout egg assembly; it accumulates and organizes yolk from precursors imported from the hemolymph and constructs a cortical cytoplasm capable, after invasion by cleavage nuclei, of generating an embryo. The cells of the follicular epithelium synthesize additional components of the yolk as well as a vitelline envelope and chorion. A low level of endopolyploidy enhances these synthetic activities. Residues of the nurse cells and follicle cells are shed at ovulation so that the oocyte is the only follicular cell remaining in the mature egg.

## Prefollicular Development

### 1. Origin of the ovarioles

A comprehensive background of ovarian structure and previtellogenic development for insects generally is provided by Büning (1994). In lepidopterans the ovaries arise as two capsules, one lying on each side of the dorsal vessel in the fifth abdominal segment. Each capsule encloses four buds of tissue, the precursors of the ovarioles. The buds remain small during the early larval instars, having a diameter of about 50 µm in the saturniid moth, *Hyalophora cecropia* (Mandelbaum 1975), but in later instars they elongate, coiling up within the capsules as they do so. Each differentiates into an apical germarium containing a mix of germ and somatic cells and a basal calyx, which will ultimately form a tube that carries ovulated eggs to an extra-ovarian oviduct.

### 2. Octet formation

After an early period of mitotic proliferation, the germ cells initiate a sequence of divisions that generate octets of cytoplasmically connected cells. Each of these differentiates during metamorphosis into seven nurse cells and an associated oocyte. The origin of insect nurse cells and their relation to the oocyte were first established in a dytiscid beetle (Giardina 1901; Gunthert 1910) and was later confirmed at the electron microscope level for *Drosophila melanogaster* (King and Büning 1985). In Dytiscids and Dipterans four successive divisions generate a bridged cluster of 16 cells but in Lepidoptera the number is uniformly eight. Formation of an octet begins when a dividing germ cell fails to complete cytokinesis. A resulting bridge of cytoplasm is stabilized by a densely staining lining, which led to its early designation as a ring canal. When a bridged pair next undergoes mitosis the two cells do so in synchrony and again fail to complete cytokinesis, thus forming a quartet of cells connected by three ring canals. Synchronous divisions occur once more and the final result is an octet interconnected by seven ring canals (Knaben 1934). Analysis of serial sections with transmission electron microscopy confirmed that in *H. cecropia* the oocyte connects directly by three ring canals to three of its siblings and only indirectly via internurse cell canals to the other four (King and Aggarwal 1965).

In *Phylosamia cynthia* (Dederer 1915), *Bombyx mori* (Yamauchi and Yoshitake 1984) and *H. cecropia* (Mandelbaum 1980) octet formation begins early in the fourth larval instar. By the end of the fifth instar, the octets lie in single file down the length of each ovariole. Sixty-eight prefollicular octets were counted in a single Feulgen-stained ovariole dissected from an early pharate adult of *Hyalophora* (King and Aggarwal 1965). In these species as well as a number of others (Grûnberg 1903) synaptonemal complexes appear in all octet nuclei, suggesting that the eight cells all initiate the first meiotic prophase during the last larval instar. At the onset of metamorphosis seven of the cells back out of meiosis (Yamauchi and Yoshitake 1984) and begin their endomitotic chromosome replications. During the two metamorphic molts, endomitosis gradually expands their DNA content and this greatly enhances the ability of nurse cells to generate RNA. DNA replication continues until mid-vitellogenesis, when the nurse cells and ring canals disintegrate. The eighth cell, the oocyte, completes meiosis much later in egg formation (Pollack and Telfer 1969).

### 3. Extrusion from the ovarian capsule

Throughout larval development four ovarioles remain coiled within each of the ovarian capsules. Oviducts formed by the imaginai disk of the external genitalia attach to the capsules and to the ends of the calyces within them. During the first metamorphic molt the calyces extrude from the capsule into the hemolymph, forming a configuration that persists during the months of pupal diapause in saturniid silkmoths. The germaria extrude during the second metamorphic molt and leave the ovarian capsule shriveled and empty in pharate adults.

The octets retain their single file arrangement in the extruded ovarioles, each with its cap of nurse cells oriented toward the apex and its oocyte toward the calyx. Tubular sleeves of somatic cells surround the octets, isolating them from the hemolymph and from contact with abdominal tracheae.

## Follicle formation

After extrusion, the file of octets and the inner sleeve of somatic cells partition to form a chain of follicles. The process begins when the sleeve moves centripetally and forms a one-cell thick epithelium, conventionally known as the follicle cells, on the surfaces of the octets. Over the nurse cells the epithelium flattens to a thickness of only a few micrometers; over the oocyte it becomes a sheet of cuboidal or columnar follicle cells (Figure 3) whose shapes change during successive stages of later development. Between octets the sleeve forms elongated, multicellular connections between successive follicles.

An outer sleeve of somatic cells differentiates during vitellogenesis into an open meshwork of longitudinal and circular striated muscle fibers (Telfer and Melius 1963) that will propel the follicles toward the calyx. Growing tracheae attach to the muscle layer, and midway through the pharate adult molt these become air-filled, thus providing for respiratory gas exchange within the ovarioles.

## Follicle dormancy

In *Hyalophora* newly formed follicles appear by several criteria to be physiologically dormant (Woodruff and Telfer 1990). Steady state membrane potentials measured between an incubation medium and the cytoplasm of the octet cells average -21 mV. Cytoplasmic pH at this time averages 6.7, close to that of the hemolymph. These values are unaffected by incubation in 10 mM azide, an inhibitor of mitochondrial ATP production, and thus appear to be maintained without ongoing support of oxidative metabolism. In newly dissected ovarioles about ten of the most recently formed follicles exhibit this state.

After about two days of dormancy, octet cell membranes hyperpolarize to average values of -45 mV and cytoplasmic pH rises to 7.4, substantially higher than the pH of the hemolymph (Woodruff and Telfer 1990). Maintenance of these values is energy-dependent but they revert to dormancy levels if 10 mM azide is added to the incubation medium. There is also a marked increase in turgor pressure at this time; dormant follicles are too flaccid to be easily impaled by capillary electrodes. Active follicles, by contrast, are visibly swollen and turgid enough to yield readily to electrode penetration.

Another crucial feature of dormancy is the absence of physiological coupling between the octet and its epithelium. When dormancy ends gap junctions become functional and allow movement of inorganic ions and other small molecules from octets to epithelia and from follicle to follicle via the interfollicular connectives (Woodruff and Telfer 1990). The end of dormancy can thus be conveniently detected by testing for electrical coupling between the octets of neighboring follicles. Shortly after the restoration of coupling light scattering particles of yolk begin to appear in the cytoplasm of the oocyte.

## Vitellogenesis

**Figure 4.**
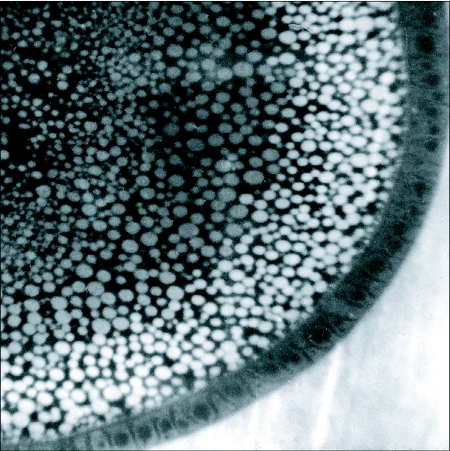
Fluorescence micrograph of a vitellogenic follicle that had been exposed *in situ* to fluorescein-labeled rabbit serum globulin. The probe had been injected into the hemolymph of a pharate adult *Hyalophora* female. Fluorescence is present in a continuous zone of protein yolk spheres that had been produced during a 24 hour incubation. The labeled yolk spheres had not mixed with the cohesive core of nonf-luorescent yolk spheres that had been produced prior to injection of the probe. The follicle was frozen and sectioned in a droplet of hemolymph whose fluorescence is visible on the lower right.

Investigations during the last century have produced a comprehensive description of the composition of yolk and the mechanism of its assembly. Like that of most other arthropods, lepidopteran yolk consists of large, protein-filled vesicles interspersed with smaller lipid droplets and particles of glycogen. In *Hyalophora* and many other silk moths the protein vesicles measure about 15 µm in diameter (Telfer 1961) and the lipid droplets about 2 µm. Protein and lipid yolk bodies form in the cortical cytoplasm of the oocyte and when completed are added to a central mass of previously deposited yolk (Figure 4), much like rings of xylem are added to the central heartwood in a growing tree.

Yolk formation is a massive process, which, over a period of several days, results in a hundreds-fold increase in the volume of each oocyte. It depletes the protein, lipid and carbohydrate reserves that are accumulated by larvae and stored in pupae. As a result, the first eggs produced by *Hyalophora* ovarioles are larger than later products (Telfer and Rutberg 1960). Large pupae that had fed well as larvae can lay several hundred more eggs than individuals that had pupated while much smaller.

### 1. The role of the nurse cells

A common earlier assumption attributed the production of yolk to the nurse cells, not withstanding the fact that the many insects that lack nurse cells also successfully store masses of proteins and lipids. The oocyte is now known to assemble yolk from products imported from the hemolymph and the follicle cells. Nurse cells function instead as generators of cytoplasm. Time course autoradiography of dipteran follicles labeled with H-uridine showed that RNA is synthesized in the endopolyploid nurse cell nuclei and moves into the cytoplasm and across the ring canals to the oocyte (Bier 1963). A similar pattern occurs in Lepidoptera (Pollack and Telfer 1969). In accord with these findings, the cytochemistry of *Hyalophora* follicles revealed dense accumulations of RNA stainable with Azure B in the cytoplasm of the nurse cells, in the cytoplasmic bridges leading to the oocyte, and in a delta of cytoplasm in the oocyte adjacent to the bridges (Figure 3). Elsewhere, small islands of RNAase-sensitive staining are dispersed among the yolk particles throughout the oocyte. About 3 µg of the nurse cell RNA are destined to be stored in each *Hyalophora* egg for use during embryo formation. The RNA content of isolated oocytes builds gradually to this level during early vitellogenesis (Pollack and Telfer 1969) and remains stabilized after the nurse cells and intercellular bridges have disintegrated. Fragmented residues of the nurse cells persist at the apical pole of the follicle until being shed at the time of ovulation.

### 2. Oocyte functions

Hybridization with 3H-polyuridylic acid indicated that nurse cell RNA moving into the oocyte includes messenger RNA (Watson et al. 1993), a fraction of which localizes in the cortical cytoplasm of the oocyte. During vitellogenesis, actin filaments maintain the integrity of the cortex. Thus, incubating vitellogenic follicles in cytocholasin D, which solubilizes actin filaments, disturbs the cytoplasmic organization of the oocyte so that the separation of cortex and yolk-filled endoplasm breaks down. The cortex is also of interest as a special region in the mature egg, because it is the site in which the blastoderm, the surface layer of cells that become the embryo and the extraembryonic membranes, will form after fertilization.

#### a. Protein uptake

**Figure 5.**
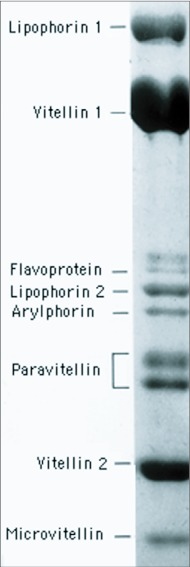
*SDS-acrylamide gel electrophoresis of yolk proteins from *Hyalophora cecropia*. The two paravitellin bands are subunits of a protein that is synthesized by ovarian follicle cells. The remaining components are fat body products that were identical to standards isolated from the hemolymph. Lipophorin bands 1 and 2 are the 250 and 85 kDa apolipophorins I and II; vitellin bands 1 and 2 are the 180 and 45 kDa subunits of vitellogenin; the flavoprotein and arylphorin bands are, 85 and 75 kDa subunits of corresponding hemolymph storage hexamers; microvitellin is a 30 kDa monomer of microvitellogenin. (Adapted from Telfer and Pan, 1988).

The protein-filled vesicles that make up most of the volume of yolk are dense enough to sediment during low speed centrifugation of saline suspensions of yolk from mature eggs. When resuspended in hypotonic media they swell and lyse and release a mix of soluble proteins, most of which are antigenically identical to proteins in the hemolymph (Telfer 1961). Literature on the biochemistry of lepidopteran yolk proteins is summarized in a general review of insect yolk proteins (Telfer 2002). The most prominent constituent is vitellogenin, a circa 500-kDa, tetrameric, female-specific lipoglycoprotein (Figure 5).

Transfer of vitellogenin from hemolymph to yolk was initially confirmed by transfusing *Antheraea polyphemus* females with hemolymph from *Hyalophora* and using species-specific antibodies to show that eggs of the recipient contained concentrates of donor vitellogenin (Telfer 1954).

Mature eggs of *Hyalophora* contain about 390 µg of vitellogenin apiece and smaller amounts of several other hemolymph-derived proteins (Figure 5) (Telfer and Pan 1988). The latter include about 86 µg of the lipid transport protein lipophorin, whose associated carotenoids give the yolk a bright yellow color, 20 µg of a 30-kDa microvitellogenin and smaller amounts of other hemolymph derivatives, including traces of several hexameric storage proteins. *Manduca sexta* adds a blue biliprotein, insectacyanin (Kang et al. 1995). The translucent chorion of this species allows the mix of carotenoids and insectacyanin to give the egg a cryptic leaf-green color.

#### b. Coated vesicles and pinosomes

**Figure 6.**
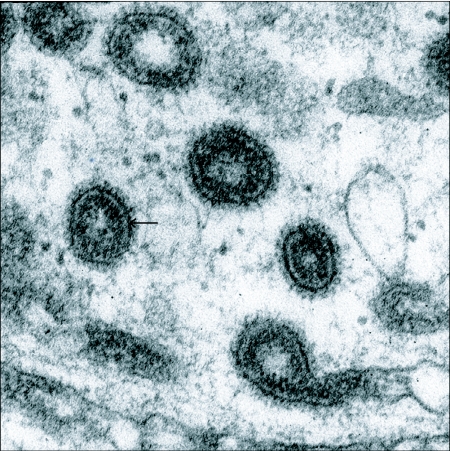
Transmission electron micrograph of endocytotic vesicles in the cortex of a vitellogenic *Hyalophora* follicle. The membrane enclosing each vesicle appears as a dark line (arrow). The structures radiating outward from the membrane are sections of the clathrin coat, a protein complex that mechanically converts a region of planar cell surface membrane into a spherical vesicle with a diameter of about 150 nm. The inner surface of each vesicle is lined by receptor-bound proteins derived from extracellular fluids. The non-staining lumen is believed to contain unbound solutes.

The import of blood proteins by animal cells is carried out by endocytosis. This is precisely the mechanism employed for protein yolk deposition by insect oocytes generally and by vitellogenic lepidopteran ovaries in particular. It begins when honeycomb-shaped patches of clathrin coat the cytoplasmic surface of the oocyte plasma membrane. When extracellular ligands such as vitellogenin bind to the outer surface of the cell membrane the patches fold inward to form circa 150 nm diameter vesicular pinosomes in the cortical cytoplasm of the oocyte (Figure 6). After entering the oocyte cortex, pinosomes shed their clathrin coats, release the adsorbed ligands to their lumina and finally transfer the resulting cargo of solutes to protein yolk bodies by membrane fusion. This sequence of configurations was first described in vitellogenic oocytes of *Aedes aegypti* (Roth and Porter 1964) and its interpretation has since been extended and validated in greater detail for this species (Raikhel 1992; Raikhel and Dhadialla 1992). Configurations consistent with the *Aedes* model are also documented for vitellogenic follicles of *Hyalophora* (Stay 1965; Telfer and Smith 1970).

Treating follicles with proton ionophores prevents the transfer of vitellogenin from endosomes to yolk bodies (Stynen et al. 1986). This effect is consistent with a general model of endosome processing in which ligands dissociate from receptors following acidification of the vesicle by a membrane proton pump. Dissociation is inhibited by proton ionophores, because they allow the captured protons to return to the cytoplasm.

#### c. Membrane turnover

Vitellogenesis entails an extraordinary rate of oocyte membrane turnover. The rate of endocytosis and the amount of cell membrane available to support it in *Hyalophora* yielded an estimate that endosome formation would exhaust the available supply of oocyte plasma membrane within a minute. A rapid recycling of processed endosomal membrane has been postulated as a way of sustaining ongoing yolk production during several days of vitellogenesis (Telfer et al. 1982). Support for this possibility comes from transmission electron microscopy, which detects tubular residues of emptied pinosomes in the oocyte cortex of vitellogenic follicles (Telfer and Smith 1970; Van Antwerpen and Law 1992).

#### d. Protein uptake kinetics

Kinetic studies of vitellogenin uptake demonstrated that *Hyalophora* follicles contain limited numbers of binding sites that can be saturated at high ligand concentrations (Kulakosky and Telfer 1987,). Half-saturation of binding sites occurred at levels close to the concentration of vitellogenin in the hemolymph; the binding mechanism thus has an affinity suitable for uptake from the hemolymph, followed by release to pinosome lumina and transfer as solutes to yolk bodies. A vitellogenin-binding protein, a putative receptor, was identified in extracts of membrane preparations of follicles from *Manduca* (Osir and Law 1986).

Lipophorin is also incorporated by a saturable mechanism (Kulakosky and Telfer 1990). Adding vitellogenin to the incubation medium suppresses the uptake of lipophorin, and to a corresponding degree lipophorin inhibits the uptake of vitellogenin. The two proteins thus either compete for a limited number of common binding sites or are bound by closely linked receptors.

The accumulation of microvitellogenin by *Hyalophora* follicles (Kulakosky and Telfer 1989) and of insectacyanin by *Manduca sexta* follicles (Kang et al. 1995) also are saturable. But since their respective uptakes are not inhibited by either vitellogenin or lipophorin, separate receptors are indicated. On the contrary, vitellogenin accelerated the rate of microvitellogenin uptake, apparently because, in addition to its receptor-bound incorporation, this protein is taken up as a solute in the fluid phase of vitellogenin endosomes, either in the lumina of the coated vesicles shown in Figure 6 or embedded in interstices of their ligand-bound lining. In confirmation of this model kinetic studies indicated that the small carbohydrate inulin is endocytosed as a solute rather than as a ligand; adding vitellogenin to the incubation medium accelerated inulin accumulation and that of microvitellogenin to equal degrees.

An earlier study using fluorescein-labeled rabbit serum globulin as a probe demonstrated transfer of fluorescence to cortical yolk spheres after injection into the hemolymph (Figure 4) (Hausman et al. 1971). Assays performed *in vitro* showed that uptake of the fluorescent probe was negligible in the absence of vitellogenin. But after addition of isolated vitellogenin to the medium fluorescent yolk was generated at a rate comparable to that during *in situ* tests. The concept that small amounts of incidental hemolymph proteins can be swept into the yolk during endocytosis of vitellogenin was thus established.

### 3. Fat body functions

#### a. Yolk protein synthesis

The fat body synthesizes and secretes into the hemolymph both vitellogenin (Pan et al. 1969) and microvitellogenin (Cole et al. 1987). Both are quantitatively female-specific and developmentally regulated proteins. The fat body also synthesizes lipophorin, which is a constituent of the hemolymph in males as well as females and at all stages of both larval development and metamorphosis (Chino et al. 1981).

#### b. Synthesis and storage of circa 500-kDa hexamers

Four widely occurring pupal storage hexamers that partition between the fat body and hemolymph of lepidopterans contribute amino acids to the synthesis of yolk proteins (Pan and Telfer 1996, Pan and Telfer 1999). Two of them accumulate primarily in crystalline protein granules that are stored in the cytoplasm of pupal fat body cells (Tojo et al. 1978; Miller and Silhacek 1982), while the other two are principal proteins of pupal hemolymph (Telfer et al. 1983; Magee et al. 1994). The two fat body storage hexamers are especially adapted for egg production. They are unique in having unusually high sulfur amino acid contents, which are useful in the production of the disulfide-reinforced chorion (Ogawa and Tojo 1981). In some species one or both of them are stored in greater amounts by female pupae than by males (Ogawa and Tojo 1981; Ryan et al. 1985; Bean and Silhacek 1988). Monarch butterflies (*Danaus plexippus*) raised in short day photoperiods retain 20–30% of their pupal stores of the high methionine hexamerins at eclosion, and these stores disappear when long day photoperiods are then used to stimulate egg production (Pan and Telfer 2001).

*Lymantna dispar* is an exceptional case because it is the one lepidopteran reported thus far that lacks the two high methionine hexamerins. In their place, female pupae store seven times greater amounts of another hexamerin, arylphorin, than males (Karpels et al. 1990).

Other pupal proteins that are hydrolyzed during pharate adult development add to the pool of amino acids that can be drawn on for egg production. This was demonstrated for *Hyalophora* pupal endocuticle, whose hydrolysis during pharate adult development releases amino acids that are absorbed from the molting fluid and incorporated into pharate adult tissues (Passonneau and Williams 1953). In addition, species that delay egg formation until after eclosion can supplement pupal stores from dietary intake and spermatophores in a variety of species-specific ways (reviewed by Ramaswamy et al. 1997). These sources are, of course, not possible in the many precocious lepidopterans whose adults do not feed and whose eggs have already ovulated by the time of eclosion.

### 4. Follicle cell functions

#### a. Intercellular spaces

As an epithelium separating the nurse cell/oocyte octet from the hemolymph, the follicle cells are in a position to regulate several aspects of egg formation. Of particular importance is a system of intercellular channels that enable hemolymph proteins to reach the oocyte by passing between cells rather than through them (Anderson and Telfer 1970). Autoradiograms of follicles that have been labeled with either 35S-sulfate or 3H-glucosamine indicate that the channels contain, in addition to hemolymph proteins, a matrix of sulfated glycosaminoglycans (Telfer 1979). The matrix can be disassembled by treating follicles with a protease under conditions that solubilize extracellular material without destroying the integrity of follicle cell or oocyte membranes. Pronase releases label from the matrix in macromolecules that span a range of sizes averaging 1-kDa. Treatment of released macromolecules with beef testis hyaluronidase yields a product that elutes in gel filtration at the same rate as an inulin standard, suggesting that a sulfated molecule equivalent in size to a trisaccharide is a monomer in the matrix carbohydrate. When vitellogenesis terminates the matrix disassembles and the channels collapse.

Vitellogenin and several other yolk precursors are more concentrated in the intercellular spaces than in the hemolymph (Anderson and Telfer 1970) and binding to the matrix is a plausible explanation of this. Since autoradiography fails to detect significant levels of labeled sulfate in yolk, protein yolk precursors must dissociate from the matrix before entering the oocyte

#### b. Secretion of yolk proteins

Autoradiograms of follicles incubated for several hours in vitro with free ^3^H-amino acids reveal concentrated labeling in newly formed yolk spheres in the cortex of the oocyte (Anderson and Telfer 1969; Melius and Telfer 1969; Ono et al. 1975). This phenomenon is seen in all lepidopterans thus far examined. It is due to follicle cell secretions that are endocytosed along with vitellogenin and other hemolymph proteins from the intercellular spaces. In most cases follicle cell products are minor constituents of the yolk relative to vitellogenin (Figure 5), but a 225-kDa, trimeric follicle cell product makes up an exceptional 25% of the protein content of the yolk in *Bombyx* (Zhu et al., 1986). Sequencing studies have identified orthologues of the *Bombyx* follicle cell product in several pyrallid species (Shirk and Perera 1998).

#### c. Trypan blue as an indicator of endocytosis

The vital dye trypan blue has a history of identifying endocytotic cells in animals and was first shown to be useful for this purpose in insects by experiments with the scorpion fly *Panorpa communis* (Ramamurty 1964). With lepidopteran ovarioles it has been useful in distinguishing vitellogenic follicles from those that have terminated hemolymph protein uptake (Figure 1) (Telfer and Anderson 1968). Within minutes the dye stains the intercellular spaces of vitellogenic follicles. With longer incubation it is endocytosed and deposited like a hemolymph protein in the yolk spheres that are forming in the oocyte cortex. Trypan blue is also useful as an indicator of cell viability for, while living cell membranes are impermeable to it, cytoplasm stains bright blue when cell membranes have been damaged. Examples can be seen in interfollicular connectives that have been handled with forceps during ovariole dissection (Figure 1).

### 5. Other activities of vitellogenic follicles

#### a. Proton pumps in Hyalophora and Manduca

Lepidopterans are generally vegetarians with diets so deficient in Na+ that the concentration of this cation in the hemolymph can be too low to drive effectively the cotransport mechanisms used by most animal cells to transport hydrophilic metabolites across their cell membranes. A remarkable remedy for this deficiency is a cell membrane proton pump that augments the equilibrium potential and yields a proton gradient that can provide the energy required for cotransport (Harvey and Wieczorek 1997). In *Hyalophora* follicles ion substitution experiments determined that this pump generates approximately 30% of the steady state potential measured between the cytoplasm of vitellogenic oocytes and a standard incubation medium (Woodruff et al. 1992). The pump is insensitive to the alkaloid ouabain, an effective inhibitor of the Na+ /K+ ATPases that drive membrane potentials in most animal cells. But it is inhibited with diagnostic sensitivity by femtamolar concentrations of bafilomycin, a fungal antibiotic that inhibits vesicular type proton ATPases (Harvey et al. 1998). The pump was isolated from the midgut epithelium of *Manduca* and found to have the subunit composition and amino acid sequences of a family of proton pumps that occur in the membranes of cytoplasmic vesicles such as pinosomes and lysosomes. Corroborating evidence of its presence in ovarian follicles was found in *Manduca*, in which antibodies to one of the subunits of the isolated midgut proton ATPase localized at the endocytotic surface of vitellogenic oocytes (Janssen et al. 1995;.

Proton V-ATPase activity has now been implicated in two activities in vitellogenic follicles. It has not only an electrogenic function unique among insects to lepidopteran cell membranes but, as noted above, is also required for endosome acidification in animals generally. The possibility thus arises that endosomal proton pumps are derived from the cell surface during vitellogenic protein uptake and then returned to the surface in empty membranes that have given up their load of vitellogenic proteins to yolk bodies.

#### b. Calcium pumps in the nurse cell cap

Steady state membrane potentials measured in the nurse cells of vitellogenic *Hyalophora* follicles are several millivolts more negative than those measured in the oocyte. When ion-selective microelectrodes were used to measure cytoplasmic levels of inorganic ions in these cells, calcium ion activities were found to be over three times higher in the oocyte than in the nurse cells (Woodruff and Telfer 1994). A calcium ion current through the intercellular bridges was inferred from these findings; it includes an electrochemical gradient down which calcium ions move from oocyte to nurse cells, replenishment of oocyte calcium ions by transmembrane leakage from the extracellular medium, and finally a pump that keeps levels low in the nurse cells by extruding calcium ions into the intercellular spaces. A variety of treatments that inhibit calcium pumps simultaneously inhibit or slightly reverse the transbridge potential (Woodruff et al. 1998).

RNA synthesis is normally undetectable by autoradiography in the vitellogenic oocyte nucleus (Pollack and Telfer 1969). But in uncoupled follicles, which lack the transbridge calcium potential uridine is incorporated by the oocyte nucleus (Woodruff and Telfer 1990, Woodruff and Telfer 1994). Transcription thus appears to be promoted by factors whose movement from the nurse cells to the oocyte is impeded by the calcium current. The transbridge potential thus reinforces the differentiation of oocytes and nurse cells with regard to their respective synthetic functions.

#### c. Lipid yolk formation

Lipids are stored in insect eggs in the protein yolk bodies as well as in the triacylglycerol droplets. The two forms are delivered to follicles by, respectively, vitellogenin and lipophorin. Vitellogenin is a dedicated protein yolk precursor, which has no other documented function in the hemolymph. It is endocytosed by the oocyte and transferred to the protein yolk bodies without detectable loss of the phospholipids and other conjugates, which are attached to it prior to its secretion by the fat body (Mundall and Law 1979).

Lipophorin, by contrast, is a multifunctional shuttle protein that carries diacylglycerols to somatic tissues such as flight muscle (Chino et al. 1981) as well as to the ovaries(Chino et al. 1977). After delivering its cargo it returns in the hemolymph for recharging by the fat body. The diacylglycerols carried to the follicle are, by inference, converted to triacylglycerols in the oocyte. Autoradiography of follicles from female *Hyalophora* injected with 3H-palmitate identified the cortical cytoplasm of the oocyte as the site of lipid droplet assembly (Wiemerslage 1976).

In saturniid moths and *Manduca* a fraction of the lipophorin reaching the oocyte is delivered to the protein yolk bodies, where it is stored along with vitellogenin after losing most of its cargo of lipids. This was first suggested in *P. cynthia*; lipophorin isolated from eggs contained 3.6% lipids while that stored in pupal hemolymph prior to egg formation has a 44% lipid content (Chino et al. 1977). Hemolymph lipophorin consists of two large apoproteins, I and II, and variable amounts of a smaller apo III, which promotes solubilization of the lipidladened form of the protein. Only apoproteins I and II are detected in the fraction of lipophorin that is stored as a yolk protein (Figure 5), dissociated apo III apparently having been returned to the hemolymph (Kawooya and Law 1988; Telfer et al. 1991).

#### d. Glycogen deposition

In insect tissues generally, glycogen is synthesized from glucose that is distributed in the hemolymph in the form of the disaccharide trehalose (Wyatt 1967). Glucose is made available for glycogen synthesis when trehalose is hydrolyzed during entry into the cell. Trehalase, the enzyme responsible for hydrolysis, is strategically located in the cytoplasmic membrane of the target cell. This model was shown to hold for the provision of glycogen precursors in ovarian follicles of *Bombyx* (Yamashita and Hasegawa 1976). *Bombyx* is of special interest here because some races enter winter dormancy after egg laying. The embryos convert stored glycogen to sorbitol and glycerol, thereby gaining a level of cryoprotection (Yamashita and Hasegawa 1985). In preparation for diapause a peptide hormone from the subesophageal ganglion stimulates the ovaries to enhance the amount of glycogen deposited in the oocyte. Diapause terminates after a period of chilling and glycogen is then regenerated from the cryoprotectants.

Autoradiography of follicles labeled *in situ* with ^3^H-glucose indicated that synthesis and deposition of glycogen are most rapid in the cortical cytoplasm of the oocyte, with the assembled particles later moving into interstices between the lipid and protein yolk bodies deeper in the oocyte. Glycogen accumulation spans a longer period than protein and lipid yolk formation: over 50% of the glycogen present in mature eggs of *Hyalophora* is deposited in the oocyte during terminal growth, a post-vitellogenic period of water uptake (Pollack 1967).

#### e. Ecdysteroid storage

Like many other insects, lepidopterans provision their eggs with high concentrations of ecdysteroids that are made available to developing embryos and prehatching larvae (reviewed in Hoffman et al. 1980). They include ecdysone and 20-hydroxyecdysone, as well as hydrophilic conjugates of these and other ecdysteroids in both *Bombyx* (Ohnishi and Chami 1977; Ohnishi et al. 1977; Hsiao and Hsiao 1979) and *Hyalophora* (Rubenstein et al. 1986). Assays of individual follicles in *Hyalophora* ovarioles showed that the ecdysteroids accumulate during both vitellogenesis and post-vitellogenic water uptake. In *Gallena mellonella* the total accumulation is equivalent to 74 µg of ecdysone per gram of eggs (Hsiao and Hsiao 1979).

#### f. Water uptake

One of the revelations of trypan blue staining was the finding that unstained follicles have up to fifty percent larger volumes than the largest staining follicles (Figure 1). This was the first indication of a terminal growth phase, which turned out to include the osmotic uptake of water (Telfer and Anderson 1968). The follicle cells swell as well as the oocyte (Wang and Telfer 1997) and this obliterates the intercellular spaces, which at this stage have lost their sulfated matrix. Studies on ion physiology demonstrated that increases in cytoplasmic concentrations of both potassium and chloride ions contribute to osmotic swelling (Wang and Telfer 1998A).

The end of water uptake can be visualized by immersing ovarioles in a hypertonic sucrose solution. Vitellogenic follicles lose water to the hypertonic medium and consequently shrivel. In *Hyalophora* approximately four terminal growth follicles also shrivel. But older follicles retain their hydrated shapes, their oocytes having been coated with a water-impermeable layer.

A water barrier makes adaptive sense in species that lay their eggs on exposed surfaces where they would quickly dehydrate in its absence. It is a temporary solution, which serves to insulate the egg until the embryo produces an even more impermeable serosal cuticle.

## Postvitellogenic development

When follicles terminate hemolymph protein uptake their intercellular spaces lose the ability to bind trypan blue and endocytosis of the dye ceases. The earlier sulfate-labeling matrix disassembles and the spaces it had occupied collapse (Telfer 1979). The follicle cells form intercellular junctions that further obstruct passage of hemolymph proteins to the oocyte. In transmission electron microscopy these sites appear similar to vertebrate tight junctions (Rubenstein 1979). In function also they resemble tight junctions; prior to their formation, fluorescent solutes injected from a capillary electrode into the space directly surrounding the oocyte exit rapidly between the follicle cells and within minutes disperse in the incubation medium. But when the tight junctions are in place, fluorescent dyes are unable to exit the follicle and seep instead under the epithelium and around the surface of the oocyte (Wang and Telfer 1997).

After the intercellular spaces have closed and hemolymph protein uptake has terminated, the follicle cells continue to secrete their yolk precursor and the oocyte continues to endocytose it. It accumulates in cortical vesicles that are smaller and denser than the earlier vitellogenin-filled yolk vesicles (Telfer and Smith 1970; Zimowska et al. 1995A, Zimowska et al. 1995B).

### 1 .Vitelline envelope growth

Throughout vitellogenesis a several µm thick vitelline envelope separates lepidopteran oocytes from their epithelia. In transmission electron micrographs the envelope has a fine, granular appearance. Although its molecular structure is not known, it is of necessity porous enough to permit passage of vitellogenin and other yolk precursors to the endocytotic surface of the oocyte. The follicle cells form villi that attach to the outer surface of the envelope while the tips of folds in the endocytotic surface of the oocyte attach to its inner surface. When follicles are briefly treated with a proteinase all attachments to the vitelline membrane loosen and it is then possible to separate intact epithelia from the oocyte manually (Anderson and Telfer 1969).

In addition, occasional villae extending from the oocyte surface penetrate the vitelline envelope and directly impinge on the inner surface of a follicle cell (Woodruff 1979). Gap junctions forming at these interfaces account for electrical coupling and the cytoplasm to cytoplasm exchanges of small molecules between oocytes and epithelia, which commence at the onset of vitellogenesis.

The vitelline envelope grows rapidly enough during vitellogenesis to cover without interruption the expanding surface area of the oocyte. When oocyte growth terminates, envelope material continues to be deposited and results in a several-fold increase in its thickness. Oocyte villi withdraw during the thickening and communication with the follicle cells through gap junctions thus terminates. Physiological isolation of the oocyte is further enforced by a layer of thin, plate-like structures, whose appearance on the outer surface of the vitelline envelope correlates with deposition of the water barrier (Telfer and Smith 1970).

The oocyte and the follicle cells now develop independently of each other. The oocyte chromosomes line up on a meiotic metaphase plate (Pollack and Telfer 1969). And ' its cortical cytoplasm reorganizes in preparation for germ band and serosa formation. The latter transformation is signaled by the swelling of the refractile bodies to low density cortical vesicles (Telfer and Smith 1970). Follicle cell products localized in these vesicles are thus made accessible for utilization early in embryogenesis as the blastoderm forms (Indrasith et al. 1987).

### 2. Chorion formation

#### a. Structure and sequential deposition

After completing the vitelline envelope the follicle cells synthesize and secrete a set of proteins that wrap the oocyte in a chorion comprising, in *Hyalophora*, approximately 50% of the dry weight of the mature egg. Though resembling insect cuticle in its mechanical properties, the chorion differs in lacking chitin and in being hardened by disulfide crosslinks between cysteine-rich fibrillar proteins, rather than by polyphenoloxidase tanning. The hatching caterpillar is able to chew a hole in the chorion as it emerges from the egg and, in fact, may ingest much of the chorion as its first meal.

Provision for sperm entry is made by a micropyle, a cluster of pores lying close to the meiotic spindle at the anterior pole of the egg. The micropyle begins to form when the ring canals disintegrate late in vitellogenesis. A dozen or so small cells congregate at this site (Pollack and Telfer 1969). A similar configuration was described in muscid fly eggs by Verhein (1921), who suggested that the micropyle results when chorion is deposited around the micropyle cells. Their withdrawal at ovulation then results in holes that will allow sperm to enter the egg. Elsewhere the chorion is laced with channels that provide for respiratory gas exchange when they fill with air after the egg is laid (Hinton 1969, Hinton 1970).

The inner layer of chorion, the first to be deposited, is a circa 0.5 µm thick aeropyle, whose porosity is maintained by a skeleton of 0.3 µm long cylindrical, trabeculae (Smith et al. 1971). A similar trabecular structure is the principal layer of the chorion in *Drosophila* (Figure 3.31b in Büning, 1994). But in lepidopterans a thick deposit of fibrous lamellae surrounds it. The structure of the lamellae has been detailed at the transmission electron level for *Hyalophora* (Smith et al. 1971) and for *A. polyphemus* (Regier et al. 1982). When deposition is complete the layer is about 60 µm thick in *Hyalophora*. In *A. polyphemus* it consists of approximately 60 lamellae.

**Figure 7.**
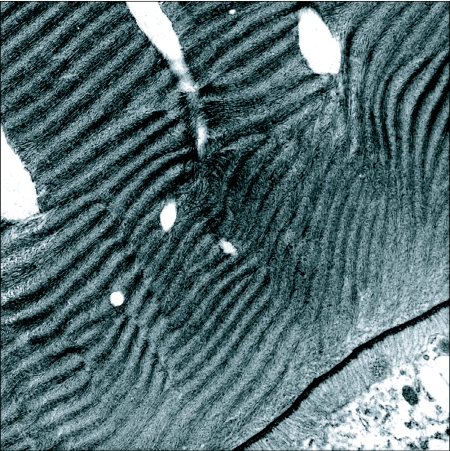
Transmission electronmicrograph of a section through the lamellar zone of a *Hyalophora* chorion. About two dozen lamellae are visible in this section. In the dark zones microfibrillae lie parallel the plane of the section. In the intervening light zones they are perpendicular to it. Late in chorion deposition localized dissolution of microfibrillae results in holes that penetrate the lamellae. These will become air-filled after the egg is laid. The secretory surface of a follicle cell is shown at the lower right corner of the figure.

Each lamella contains multiple layers of microfibrillae (Figure 7) that lie parallel to the surface of the egg. Within a lamella the orientation of successive strata of microfibrillae varies relative to the longitudinal axis of the egg from parallel to circular. The arrangement endows the chorion with multidirectional tensile strength. The succession of orientations results in a characteristic alternation of dark and light zones in sectioned chorions (Figure 7). In arthropod cuticle also such patterns suggest multidirectional tensile strength, though in that case chitin is a principal component (Neville 1967).

While depositing the chorion the follicle cells remain a single cell thick epithelium and within that layer have a close-packed hexagonal arrangement. At each tripartite cellular junction resulting from this arrangement the three cells give rise to a cluster of villi that extend through the underlying lamellae and terminate on the trabecular aeropyle. Late in lamella deposition the cluster of villi becomes a focus of microfibril dissolution; the result is a several µm diameter radial canal that connects the surface of the chorion to the inner aeropyle.

The lamellar layer undergoes modifications after its deposition has been completed. The microfibrillae thicken and fuse in a way that ultimately obscures the lamellae. Their initial arrangement becomes disrupted by a disorderly array of channels. As the channels form fine filaments run though them lengthwise, whose timing and arrangement suggest a role in guiding channel formation. The initial deposition and subsequent modification of the lamellae are remarkable examples of the self-assembly of secreted proteins into a complex extracellular structure.

After completion and modification of the lamellar layer cylinders of spines form on the surface of the *Antheraea* chorion, one around each of the tripartite aeropyle channel openings. The presence and distribution of these crowns vary widely between species. They cover the entire surface of the egg except the micropyle in many species of *Antheraea*, but are restricted to circumferential bands in others and are altogether absent from many silkmoths, including *Bombyx* and *Hyalophora*. Deposition begins when lamella formation reinitiates and is completed when filler proteins invade the new lamellae (Regier et al. 2005.) Phylogenetic analysis led to a model in which aeropyle crowns are proposed to have arisen just once during the evolution of *Antheraea* and to have undergone several subsequent reductions in distribution.

During deposition the chorions of *Antheraea* and *Hyalophora* are transparent enough to reveal the yellow pigments deposited in the yolk they enclose. When the egg dries after being laid, however, the channels of the trabecular and lamellar layers fill with air and the chorion becomes opaque.

#### b. Chorion proteins encoded by multigene families

In the 1970's the deposition of silkmoth chorions became focal objects for pioneering studies on the roles of genes and proteins in morphogenesis. A detailed review and bibliography are provided by Regier and Kafatos (1985), in whose laboratory these studies were done. Some of their major conclusions are summarized here to illustrate the scope and complexity of the machinery employed by the follicular epithelium as it generates this extraordinary structure.

Two-dimensional gel electrophoresis identified a total of 186 polypeptides in extracts of the Antheraea chorion. These clustered mainly in three groups with molecular weights of 9–12, 12–14 and 16–20 kDa. For convenience of discussion the three were designated as groups A, B and C, respectively. That the 186 proteins are original translates rather than products of the subdivision of longer translates was indicated in several ways, including translation of follicle cell RNA in a cell-free wheat germ extract. The products produced the same 2-D gel electrophoresis pattern as extracts of chorion, with the exception that their positions were shifted toward larger sizes due to the presence of signal polypeptides that are normally cleaved during secretion.

Amino acid sequences of partial protein fragments as well as those deduced from base sequences of cDNA and chromosomal clones of *Antheraea* clarified relationships among 128 of these proteins. Group A protein amino acid sequences averaged close to 98% identity and were therefore considered to be products of a closely related family of genes. Two subfamilies within this group differed from each other by the presence or absence of specific sequence gaps. Proteins in the B group exhibited similarly close sequence identity but were less closely related to the A proteins. B proteins too divided into two subfamilies. The family and subfamily relations imply a history of many successive gene duplications and subsequent divergence by mutation into the proteins that assemble in contemporary chorions.

The availability of mutant strains permitted localization of chorion genes by recombination techniques on the twenty-four chromosomes of *Bombyx* (Goldsmith and Basehoar 1978). Chorion genes were found in three clusters, all at one end of the second chromosome.

Predictions of secondary structure from amino acid sequences indicated a tripartite structure in the three A, B and C groups. A third to a half of the amino acids are in an evolutionarily conserved central region. The central region has a high content of the nonpolar amino acids glycine, alanine, valine and leucine, which permit it to participate in β-sheet formation as the proteins condense to form the microfibrillae seen in electron micrographs. The carboxyl and amino terminal arms, by contrast, contain greater sequence variability, have higher polar amino acid contents and thus are presumed to be more exposed to the aqueous interstices of the developing chorion (Regier and Kafatos, 1985).

#### c. Time sequences in chorion protein synthesis

The arrangement of follicles in a developmental sequence within each ovariole facilitated detection of the times of synthesis and the sites of deposition of the 186 proteins of the *Antheraea* chorion. Thus, during each of five phases of chorion formation (trabecular layer deposition, the initial formation of the lamellar layer, expansion and then densification of the lamellar layer and finally the formation of the aeropyle crowns that cap the outer surface of the mature chorion in this species) a unique set of proteins is synthesized. Short term labeling in organ cultures followed by two-dimensional gel analysis allowed further resolution into 10 successive synthetic profiles, each accounting for about 10% of the final mass of the chorion. The second through ninth periods each lasted about 3 hours. The first and tenth were further subdivided into additional 3 hour periods entailing the synthesis of smaller amounts of protein. Peak rates of synthesis of individual proteins were generally confined to one or two successive time periods. Classification of the A and B families into two subfamilies also has developmental significance, as in both cases the members of one subfamily were synthesized during middle periods of chorion deposition and of the other subfamily during late deposition. Formation of the chorion thus involves a stepwise series of changes in the identities of the proteins being synthesized.

Cloning techniques also established for several A and B proteins that during peak periods of synthesis maximum levels of a corresponding messenger RNA were also present. Regulation of transcriptional activity is thus a potential mechanism for generating the stepwise changes in chorion protein synthesis.

Newly synthesized proteins that have been labeled during incubation of follicles in radioactive amino acids are soluble in aqueous buffers, but three hours later they require denaturing agents for extraction. The transition is consistent with their being secreted as solutes that can diffuse into the developing chorion but that then condense by hydrophobic bonding to form the microfibrillae.

After ovulation an additional change in solubility occurs. Prominent among the hydrophilic amino acids of the carboxyl- and amino-terminal arms are cysteine residues, most of which remain in the reduced state during chorion deposition. When the egg separates from its follicular epithelium during ovulation, however, they participate in disulfide bond formation and this results in hardening of the chorion. Denaturing agents no longer suffice to solubilize the chorion but must be supplemented with disulfide reducing agents. The egg is now prepared for survival in the environment, after being fertilized and glued to a substratum.

## Control of follicle development

### 1. Hormonal controls

A study on the control of vitellogenesis in the army worm *Spodoptera frugiperda* provided a simple and convincing confirmation that juvenile hormone and 20-hydroxyecdysone, can both affect the timing of ovarian development in a lepidopteran (Sorge et al. 2000). Vitellogenin synthesis begins in this species about five hours after eclosion and yolk is visible in cytoplasm of oocytes three or four days later. Females decapitated immediately after eclosion produced neither vitellogenin nor yolk, but could be rescued from these deficits by injections of juvenile hormone. Injection of 20-hydroxyecdysone also promoted vitellogenin synthesis in decapitated females, but the oocytes failed to deposit yolk unless juvenile hormone or its substitute methoprene was also administered. Juvenile hormone not only enhanced the ability of follicles to incorporate vitellogenin into cytoplasmic yolk but also stimulated them to secrete 20-hydroxyecdysone.

#### a. Juvenile hormone

Juvenile hormone promotes reproduction in a wide variety of insects (reviewed by Wyatt and Davey 1996). The roles played by this versatile hormone vary widely, but as in *Spodoptera* promoting the synthesis of vitellogenin is prominent among them (Pan and Wyatt 1971; Ramaswamy et al. 1997; Zeng et al. 1997). Thus, Monarch butterflies raised under short day conditions eclose with a hemolymph that lacks detectable vitellogenin and with oocytes that lack yolk. These activities can be triggered even in short day females, however, by injections of juvenile hormone.

By contrast, juvenile hormone is not required for vitellogenesis in precocial lepidopterans that eclose with oviducts already full of ovulated eggs. In *Hyalophora* for instance vitellogenin first appears in the hemolymph during the larval/pupal molt and its concentration remains high in pupae and pharate adults until being drawn down by transfer to the oocytes during vitellogenesis (Telfer 1954). Vitellogenin synthesis and egg production both proceed on this schedule in *Hyalophora* in the absence of corpora allata and of juvenile hormone (Pan 1977).

*Manduca* is an intermediate case. Traces of vitellogenin may be present in the hemolymph of prepupae (Satyanarayana et al. 1994), but the peak of synthesis associated with vitellogenesis does not begin until several days prior to adult eclosion (Imboden and Law 1983). Yolk deposition begins as the females eclose (Nijhout and Riddiford 1974). In females allatectomized as pupae, vitellogenin synthesis and yolk production both occur on this schedule. But within a week yolk-filled oocytes begin to disintegrate and the follicles never complete the assembly of eggs. In *Manduca* as well as in two species of *Chonstoneura* (Delisle and Cusson 1999) only the postvitellogenic stages of egg formation require the presence of juvenile hormone.

#### b. Ecdystereroids

That 20-hydroxyecdysone directly or indirectly triggers aspects in ovarian development becomes apparent during the metamorphic molts. Differentiation of octets into oocytes and nurse cells (Orikasa et al. 1993), extrusion of calyces from the ovarian capsules and synthesis of vitellogenin (Pan, 1977) are among the changes triggered by the hormonal regimen responsible for the first metamorphic molt. The final differentiation of pupal ovaries to a stage that is competent to initiate vitellogenin uptake occurs in *Hyalophora* (Williams 1952) and *Bombyx* (Ohnishi 1987) during the second metamorphic molt, again implying promotion by 20-hydroxyecdysone and the potential for inhibition by juvenile hormone. Ironically, 20-hydroxyecdysone can also block egg formation. Having successfully triggered ovarian differentiation in *Plodia interpunctella* pupae, it then becomes an inhibitor of vitellogenesis: yolk fails to appear in oocytes and eggs are not produced by females that are injected with 20-hydroxyecdysone late in adult development (Shirk et al. 1990).

The detection of putative ecdysone receptors in ovarian tissue reinforces the evidence for hormonal control. The technology leading to receptor detection has been adopted from studies of mammalian insulin-like proteins and their corresponding receptors (reviewed for a wide variety of animals by Wu and Brown 2006). Isolation and molecular analyses of neurosecretory products from *Bombyx* identified a prothoracico tropic hormone, now known as bombyxin, that activates ecdysteroid secretion during molting and metamorphosis. Bombyxin turned out to belong to a large family of proteins with amino acid compositions and sequences closely resembling the vertebrate peptide hormone insulin. Insulin receptors are another well characterized family of proteins; a 178 kDa protein has been precipitated from several tissues of *Manduca* by antibodies to human insulin receptor (Smith et al. 1998).

Ovarian tissues from several Lepidoptera have been shown to bind bombyxin (Fullbright et al. 1997). Thus, chemically synthesized and labeled bombyxin bound specifically to isolated membrane preparations of prepupal, pupal and adult ovaries from *B. mori* and *Samia cynthia* and to an ovarian cell line from *Spodoptera*. The latter binding had a dissociation constant of 260 picomolar and the receptor had a subunit structure resembling that of mammalian insulin receptors.

An example of the function of insulin receptor-like proteins in an insect ovary is provided by studies on chico, an insulin receptor-like protein from *Drosophila*. In females homozygous for a chico mutation proliferation of follicle cells was suppressed and follicles failed to undergo vitellogenesis (Drummand-Barbosa and Spradling 2001). Since wild type ovaries transplanted to homozygotes were able to produce yolk (Richard et al. 2005), the mutant does not result in a lack of circulating vitellogenic hormones, but acts autonomously within the ovary.

Adding to the complexity of ecdysteroid effects on vitellogenesis is the extraordinary amounts of ecdysteroids found in ovarian follicles. In *Bombyx* ecdysteroids are present in normal amounts in isolated female abdomens but are not present in either males or in ovariectomized females (Hanaoka and Ohnishi, 1974). They thus appear to be products of the ovaries themselves rather than imported products of the prothoracic glands.

#### c. Absence of evidence for female reproductive hormones

In a pioneering study on lepidopteran endocrinology, Kopec (1911) concluded that female-specific hormones are not required for the development of follicles, for pupal ovaries implanted into males successfully assembled and ovulated eggs. In a wide variety of other lepidopterans tested over the last century male hemolymph has shown a similar capacity (Lamy 1979). There are, albeit, some deficiencies in male-grown eggs: they are smaller than those produced in females, but this results in part from a lack of vitellogenin in male hemolymph (Telfer and Rutberg 1960). And in *Hyalophora* the synthetic functions of follicle cells are quantitatively reduced, with male-grown eggs containing only 54% as much chorion and 33% as much endogenously synthesized yolk protein as eggs produced in females (Pan et al. 1994). But even these deficiencies do not interfere with the sequence of follicular transitions required to make an egg, for in *Bombyx* the nurse cells produced normal amounts of egg RNA and larvae were able to hatch from parthenogenetically activated male-grown eggs (Yamashita and Irae 1980).

#### d. Cyclic nucleotide inhibition of patency

The termination of vitellogenesis, a key step in late follicular development, normally occurs in *Hyalophora* follicles when they have reached a length of 2 mm. But incubation in membrane-permeable analogues of cyclic adenosine monophosphate (cAMP) can induce the response in any vitellogenic follicle, regardless of its size (Wang and Telfer 1996). The response is due to closure of the intercellular spaces (Wang and Telfer 1997). Synthesis of the sulfated glycosaminoglycans deposited in the intercellular channels of the follicular epithelium is inhibited, water uptake causes the follicle cells to swell and close the emptied channels and tight junctions form between neighboring follicle cells. As noted above, these are exactly the changes exhibited by follicle cells during *in situ* termination of vitellogenesis.

A common mechanism of cyclic nucleotide control of physiological activities in animal cells is by activation of a protein kinase A, which transfers phosphate from ATP to other proteins, including ion channels, receptors and other kinases, and so can initiate a cascade of protein phosphorylations. Pharmacological approaches detected just such a reaction involved in the termination of vitellogenin uptake. Inhibition of protein kinase A protected the follicle from the effects of cAMP. Activation of protein kinase A by other routes imitated the effects of cAMP (Wang and Telfer 1996). Since over 80% of the protein kinase A activity in a follicle is located in the follicle cells (Wang and Telfer, 2000) it is appropriately located for transforming this porous layer of cells into a tight epithelium.

Follicles can also be made to terminate vitellogenin uptake by incubation in pertussis toxin (Wang and Telfer 1998B). This too is mediated by conversion of the follicle cells to a tight epithelium. Pertussis toxin is an adenyl ribosyl transferase, whose substrate is the a_i/o_-subunit of trimeric G proteins that couple to transmembrane receptors and are widely employed by animal cells in their responses to hormones. Homologues of a mammalian _ai/o_ subunit were identified in immunoblots of *Hyalophora* follicle cell membrane extracts.

The effects of cAMP, protein kinase A and pertussis toxin combine to demonstrate that the elements of a signal transduction system capable of terminating patency are present in vitellogenic follicles. But a change in response to this control system occurs abruptly when follicles reach a length of about 2.0 mm. These follicles are bathed by the same hemolymph that supports ongoing patency in younger follicles and their transformation could well result from a developmetal restructuring of the signal tranduction system. The possibilities include addition or modification of a receptor and a change in the availability of an intrafollicular ligand. The example reinforces the principle that transformations in follicular function are responses to an internal program of development, however much they may also require the presence of hemolymph factors such as juvenile hormone, ecdyteroids, vitellogenin and other activating ligands.

A later study on cyclic nucleotide control of patency yielded very different results in *Heliothis virescens* (Pszczolkowski et al. 2005). In this species activation of protein kinase A with a cell-permeant analogue of cAMP induced shrinkage of the follicle cells and hence apparently rendered the epithelium morphologically patent, though whether the effect went so far as to promote vitellogenin uptake was not measured. Egg production in *Heliothis* is promoted by juvenile hormone (Shu et al.) and, accordingly, incubation in juvenile hormone I also promoted patency. That protein kinase activity should facilitate patency in *Heliothis* while inhibiting it in *Hyalophora* is clearly correlated with differences between these two species in the timing and dependence of vitellogenesis on juvenile hormone. The *Heliothis* case is further complicated by the finding that juvenile hormone III promotes morphological patency via activation of another kinase system passing through protein kinase C. It would be interesting to know how patency is controlled in a species such as *Manduca*, in which juvenile hormone promotes postvitellogenic growth of the follicle, rather than vitellogenesis.

## Predictions

Predicting the future of research on lepidopteran egg formation is not possible. Examples of areas in which promising starts have already been made but which need still more detailed study include interactions between epithelial and octet cells, proton physiology, hormone dependence, cyclic nucleotide-based transduction systems, and the ever-elusive intrinsic mechanisms of developmental control. But which if any of these will attract further investigation? Funding opportunities and unforeseen advances in general biology and entomology as well as opportunities due to new laboratory techniques may tend to divert attention from older questions.

Finally, as in the past, new insights will be guided by the insects that become popular for laboratory studies. Before the miniaturization of molecular techniques late in the last century, giant silkmoths such as *Hyalophora*, the domestic silkworm *Bombyx* and later the sphingid *Manduca* were among the species providing the hemolymph and yolk proteins that catalyzed studies of egg formation. Hand dissections of these large moths uncovered in single ovarioles eye-opening, step-wise sequences in morphogenesis and these also became powerful stimuli for investigations of egg formation. What new species and observations will stimulate further studies in this area?

## References

[bibr01] Anderson L, Telfer W (1969). A follicle cell contribution to the yolk spheres of moth oocytes.. *Tissue and Cell*.

[bibr02] Anderson L, Telfer W (1970). Extracellular concentrating of proteins in the Cecropia moth follicle.. *Journal of Cell Physiology*.

[bibr03] Bean D, Silhacek D (1988). Changes in titer of the female-predominant storage protein (81K) during larval and pupal development of the wax moth, *Galleria mellonella*.. *Archives of Insect Biochemistry and Physiology*.

[bibr04] Bier K (1963). Synthesis, intercellular transport and decomposition of RNA in the ovary of *Musca domestica*.. *Journal of Cell Biology*.

[bibr05] Büning J (1994). *The Insect Ovary.*.

[bibr06] Cardoen J, Watson C, DeLoof A, Berry S (1990). Polyploidy in the nuclei of ovarian nurse and follicle cells of the silk moth, *Hyalophora*.. *Archives of Insect Biochemistry and Physiology*.

[bibr07] Chino H, Downer R, Takahashi K (1977). The role of diacylglycerol-carrying lipoprotein I in lipid transport during insect vitellogenesis.. *Biochimica et Biophsyica Acta*.

[bibr08] Chino H, Downer R, Wyatt G, Gilbert L (1981). Lipophorin, a major class of lipoproteins of insect hemolymph.. *Insect Biochemistry*.

[bibr09] Cole K, Boduski G, Fernando-Warnakulasuriya G, Freeman M, Gordan J, Clark W, Law J, Wells M (1987). Primary structure and comparative sequence analysis of an insect apoliproprotein.. *Journal of Biological Chemistry*.

[bibr10] Dederer P (1915). Oogenesis in *Philosamia cynthia*.. *Journal of Morphology*.

[bibr11] Delisle J, Cusson M (1999). Juvenile hormone biosynthesis, oocyte growth and vitellogenin accumulation in *Choristoneura fumiferana* and *C. rosaceana*: a comparative study.. *Journal of Insect Physiology*.

[bibr12] Drummond-Barbosa D, Spradling A (2001). Stem cells and their progeny respond to nutritional changes during *Drosophila* oogenesis.. *Developmental Biology*.

[bibr13] Fullbright G, Lacy E, Büllesbach E (1997). The prothoracicotropic hormone bombyxin has specific receptors on insect ovarian cells.. *European Journal of Biochemistry*.

[bibr14] Giardina A (1901). Origine dell' oocite e delle cellule nutrici nei *Dytiscus*.. *Internationale Monattschrift für Anatomie und Physiologie*.

[bibr15] Goldsmith M, Basehoar G (1978). Organization of chorion genes in *Bombyx mori*, a multigene family. I. Evidence for linkage to chromosome 2.. *Genetics*.

[bibr16] Grünberg K (1903). Keim und Nahrzellen in den Hoden und Ovarien der Lepidotera.. *Zeitschrift für wissenschafiliche Zoologie*.

[bibr17] Gunthert T. (1910). Die Eibildung der Dytisciden.. *Zoologische Jahrbuch, Abteilung für Anatomie und Ontologie*.

[bibr18] Hanaoka K, Ohnishi E (1974). Changes in ecdysone titre during pupaladult development in the silkworm, *Bombyx mori*.. *Journal of Insect Physiology*.

[bibr19] Harvey W, Maddrell S, Telfer W, Wieczaorek H (1998). H+ ATPases energize animal plasma membranes for secretion and absorption of ions and fluids.. *American Zoologist*.

[bibr20] Harvey W, Wieczorek H (1997). Animal plasma membrane energization by chemiosmotic proton V-ATPases.. *Journal of Experimental Biology*.

[bibr21] Hausman S, Anderson L, Telfer W (1971). The dependence of Cecropia yolk formation in vitro on specific blood proteins.. *The Journal of Cell Biology*.

[bibr22] Hinton H (1969). Respiratory systems of insect egg shells.. *Annual Review of Entomology*.

[bibr23] Hinton H (1970). Insect egg shells.. *Scientific American*.

[bibr24] Hoffman J, Lageaux M, Hertru C, Charlet M, Goltzene F, Hoffman J (1980). Ecdysone in reproductively competent female adults and embryos in insects.. *Progress in Ecdysone Research*.

[bibr25] Hsiao T, Hsiao D (1979). Ecdysteroids in the ovary and eggs of the greater wax moth.. *Journal of Insect Physiology*.

[bibr26] Imboden H, Law J (1983). Heterogeneity of vitelline and vitellogenin of the tobacco hornworm, *Manduca sexta* L. Time course of vitellogenin appearance in the haemolymph of the adult female.. *Insect Biochemistry*.

[bibr27] Indrasith L, Furusawa T, Shikata M, Yamashita O (1987). Limited degradation of vitellin and egg-specific protein in *Bombyx* eggs during embryogenesis.. *Insect Biochemistry*.

[bibr28] Janssen I, Hendrickx K, Klein U, DeLoof A (1995). Immunolocalization of a proton V-ATPase in ovarian follicles of the tobacco hornworm *Manduca sexta*.. *Archives of Insect Biochemistry and Physiology*.

[bibr29] Kang Y, Kulakosky P, Van Antwerpen R, Law J (1995). Sequestration of insectacyanin. a blue hemolymph protein, into the eggs of the hawkmoth, *Manduca sexta*. Evidence for receptor-mediated endocytosis.. *Insect Biochemistry and Molecular Biology*.

[bibr30] Karpels S, Leonard D, Kunkel J (1990). Cyclic fluctuations in arylphorin, the principal serum storage protein of *Iymantria dispar*, indicate multiple roles in development.. *Insect Biochemistry*.

[bibr31] Kawooya J, Law J (1988). Uptake of the major hemolymph lipoprotein and its transformation in the insect egg.. *Journal of Biological Chemistry*.

[bibr32] King R, Aggarwal S (1965). Oogenesis in *Hyalophora cecropia*.. *Growth*.

[bibr33] King R, Büning J, Kerkut G, Gilbert L (1985). The origins and functions of insect oocytes and nurse cells.. *Comprehensive Insect Physiology, Biochemistry and Pharmacology*.

[bibr34] Knaben N (1934). Oogenese bei *Tischeria angusticolella*.. *Zuschrift für Zellforschung und mikroskopische Anatomie*.

[bibr35] Kopec S (1911). Untersuchungen uber Kastration und Transplantation be Schmetterlingen.. *Roux's Archive für Entwicklungsmechanic*.

[bibr36] Kulakosky P, Telfer W (1987). Selective endocytosis, *in vitro*, by ovarian follicles from *Hyalophora cecropia*.. *Insect Biochemistry*.

[bibr37] Kulakosky P, Telfer W (1989). Kinetics of yolk precursor uptake in *Hyalophora cecropia*: stimulation of microvitellogenin endocytosis by vitellogenin.. *Insect Biochemistry*.

[bibr38] Kulakosky P, Telfer W (1990). Lipophorin as a yolk precursor in *Hyalophora cecropia*: uptake kinetics and competition with vitellogenin.. *Archives of Insect Biochemistry and Physiology*.

[bibr39] Lamy M (1979). Vitellogenese proteique en mileau male chez les Lepidopteres.. *Annales Biologique Francaise*.

[bibr40] Magee J, Kraynack N, Massey H, Telfer W (1994). Properties and significance of a riboflavin-binding hexamerin in the hemolymph of *Hyalophora cecropia*.. *Archives of Insect Biochemistry and Physiology*.

[bibr41] Mandelbaum I (1975). Intercellular Bridge Development during Gametogenesis in *Hyalopohora cecropia*..

[bibr42] Mandelbaum I (1980). Intercellular bridges and the fusome in the germ cells of the cecropia moth.. *Journal of Morphology*.

[bibr43] Melius M, Telfer W (1969). An autoradiographic analysis of yolk deposition in the cortex of the Cecropia moth oocyte.. *Journal of Morphology*.

[bibr44] Miller S, Silhacek D (1982). The synthesis and uptake of haemolymph storage proteins by the fat body of the greater wax moth, *Galleria mellonella* (L.).. *Insect Biochemistry*.

[bibr45] Mundall E, Law J (1979). Physical and chemical characterization of vitellogenin from the hemolymph and eggs of the tobacco hornworm, *Manduca sexta*.. *Comparative Biochemistry and Physiology*.

[bibr46] Neville A (1967). Chitin orientation in cuticle and its control.. *Advances in Insect Physiology*.

[bibr47] Nijhout M, Riddiford L (1974). The control of maturation by juvenile hormone in the tobacco hornworm moth *Manduca sexta*.. *Biological Bulletin*.

[bibr48] Ogawa K, Tojo S (1981). Quantitative changes of storage proteins and vitellogenin during the pupal-adult development in the silkworm, *Bombyx mori* (Lepidoptera:Bombycidae).. *Applied Entomology and Zoology*.

[bibr49] Ohnishi E (1987). Growth and maturation of ovaries in isolated abdomens of *Bombyx mori*: responses to ecdysone and other steroids.. *Zoo logical Science*.

[bibr50] Ohnishi E, Chami F (1977). Biosynthesis of ecdysone in the isolated abdomen of the silkworm, *Bombyx* mori.. *Development, Growth and Differentiation*.

[bibr51] Ohnishi E, Mizuno T, Chataani F, Ikekawa M, Sakurai S (1977). 2-deoxy-alpha-ecdysone from ovaries and egg of the silkworm *Bombyx mori*.. *Science*.

[bibr52] Ono S, Nagayama H, Shimura K (1975). The occurrence and synthesis of female- and egg-specific proteins in the silkworm, *Bombyx mori*.. *Insect Biochemistry*.

[bibr53] Orikasa C, Yamauchi H, Nagasawa H, Suzuki H, Nagata M (1993). Induction of oocyte-nurse cell differentiation in the ovary by the brain during the initial stage of oogenesis in the silkworm *Bombyx mori* (Lepidoptera: Bombycidae).. *Applied Entomology and Zooligy*.

[bibr54] Osir E, Law J (1996). Specific binding and uptake of 125I-labelled vitellogenin by follicles of the tobacco hornworm, *Manduca sexta*.. *Archives of Insect Biochemistry and Physiology*.

[bibr55] Pan M (1977). Juvenile hormone and vitellogenin synthesis in the Cecropia silkworm.. *Biological Bulletin*.

[bibr56] Pan M, Bell W, Telfer W (1969). Vitellogenic blood protein synthesis by insect fat body.. *Science*.

[bibr57] Pan M, Wyatt G (1971). Juvenile hormone induces vitellogenin synthesis in the monarch butterfly.. *Science*.

[bibr58] Pan M, Wiemerslage L, Telfer W (1994). Male-grown eggs in *Hyalophora*: deficient follicle cell secretion as well as protein and lipid yolk deposition.. *Journal of Insect Physiology*.

[bibr59] Pan M, Telfer W (1996). Methionine-rich hexamerin and arylphorin as precursor reservoirs for reproduction and metamorphosis in female luna moths.. *Archives of Insect Biochemistry and Physiology*.

[bibr60] Pan M, Telfer W (1999). Equivalence of riboflavin-binding hexamerin and arylphorin as reserves for adult development in two saturniid moths.. *Archives of Insect Biochemistry and Physiology*.

[bibr61] Pan M, Telfer W (2001). Storage hexamer utilization in two lepidopterans: differences correlated with the timing of egg formation.. *Journal of Insect Science*.

[bibr62] Passonneau J, Williams C (1953). The moulting fluid of the cecropia *silkworm*.. *Journal of Experimental Biology*.

[bibr63] Pollack S (1967). *Synthisis and distribution of RNA in Cecropia oocyte differentiation Ph D Dissertation.*.

[bibr64] Pollack S, Telfer W (1969). RNA in Cecropia moth ovaries: sites of synthesis, transport and storage.. *Journal of Experimental Zoology*.

[bibr65] Pszczolkowski M, Peterson A, Srinivasan A, Ramaswamy S (2005). Pharmacological analysis odf ovarial patency in *Heliothis virescens*.. *Journal of Insect Physiology*.

[bibr66] Raikhel A (1992). Vitellogenesis in mosquitoes.. *Advances in Disease Vector Research*.

[bibr67] Raikhel A, Dhadialla T (1992). Accumulation of yolk proteins in mosquito oocytes.. *Annual Review of Entomology*.

[bibr68] Ramamurty P (1964). On the contribution of the follicle epithelium to the deposition of yolk in the oocyte of *Panorpa communis*.. *Experimental Cell Research*.

[bibr69] Ramaswamy S, Shu S, Park Y, Zeng F (1997). Dynamics of juvenile hormone-mediated gonadotropism in the Lepidoptera.. *Archives of Insect Biochemistry and Physiology*.

[bibr70] Regier J, Mazur G, Kafatos F, Paul M (1982). Morphogenesis of silkmoth chorion: initial framework formation and its relation to specific proteins.. *Developmental Biology*.

[bibr71] Regier J, Kafataos F, Kerkut G, Gilbert L (1985). Molecular aspects of chorion formation.. *Comprehensive Insect Physiology, Biochemistry and Pharmacology*.

[bibr72] Regier J, Paukstadt U, Paukstadt L, Mitter C, Peigler R (2005). Phylogenetics of eggshell morphogenesis in *Antheraea* (Lepidoptera: Saturniidae): unique origin and repeated reduction of the aeropyle crown.. *Systematic Biology*.

[bibr73] Richard D, Rybczynski R, Wilson T, Wang Y, Wayne M, Zou Y, Harshman L (2005). Insulin signaling is necessary for vitellogenesis in *Drosophila melanogaster* independent of the roles of juvenile hormone and ecdysteroids: female sterility of the chico insulin signaling mutation is autonomous to the ovary.. *Journal of Insect Physiology*.

[bibr74] Roth T, Porter K (1964). Yolk protein uptake in the oocyte of the mosquito, *Aedes aegypti* L.. *Journal of Cell Biology*.

[bibr75] Rubenstein E (1979). The role of an epithelial occlusion zone in the termination of vitellogenesis in *Hyalophora cecropia* ovarian follicles.. *Developmental Biology*.

[bibr76] Rubenstein E, Kelly T, Culbert C, Woods C, Weeks B (1986). Ecdysteroids in developing ovarian follicles of *Hyalophora cecropia*.. *Archives of Insect Biochemistry and Physiology*.

[bibr77] Ryan R, Keim P, Wells M, Law J (1985). Purification and properties of a predominantly female-specific protein from the hemolymph of the larva of the tobacco hornworm, *Manduca sexta*.. *Journal of Biological Chemistry*.

[bibr78] Satyanarayana K, Bradfield J, Bhaskaran G, Dahm K (1994). Stimulation of vitellogenin production by methoprene in prepupae and pupae of *Manduca sexta*.. *Archives of Insect Biochemistry and Physiology*.

[bibr79] Shirk P, Bean D, Brookes V (1990). Ecdysteroids control vitellogenesis and egg maturation in pharate adult females of the Indian meal moth *Plodia interpunctella*.. *Archives of Insect Biochemistry and Physiology*.

[bibr80] Shirk P, Perera O (1998). 5'coding region of the follicular epithelium yolk polypeptide 2 cDNA in the moth *Plodia interpunctela* contains an extended coding region.. *Archives of Insect Biochemistry and Physiology*.

[bibr81] Shu S, Park S, Ramaswamy S, Srinivasan A (1998). Temporal profiles of juvenile hormone titers and egg production in virgin and mated females of *Heliothis virescen*.. *Journal of Insect Physiology*.

[bibr82] Smith D, Telfer W, Neville A (1971). Fine structure of the chorion in a moth, *Hyalophora cecropia*.. *Tissue and Cell*.

[bibr83] Smith W, Koundinya M, McAllister T, Brown A (1998). Insulin receptor-like tyrosine kinase in the tobacco hornworm, *Manduca sexta*.. *Archives of Insect Biochemistry and Physiology*.

[bibr84] Sorge D, Nauen R, Range S, Hoffmann K (2000). Regulation of vitellogenesis in the fall armyworm *Spodoptera frugiperda fapidoptera: Noctuidae*).. *Journal of Insect Physiology*.

[bibr85] Stay B (1965). Protein uptake in the oocytes of the Cecropia moth.. *Journal of Cell Biology*.

[bibr86] Stynen D, Woodruff R, Telfer W (1988). Effects of ionophores on vitellogenin uptake by *Hyalophora* oocytes.. *Archives of Insect Biochemistry and Pysiology*.

[bibr87] Telfer W (1954). Immunological studies of insect metamorphosis II: the role of a sex-limited blood protein in egg formation by the cecropia silkworm.. *Journal of General Physiology*.

[bibr88] Telfer W (1961). The route of entry and localization of blood proteins in the oocytes of saturniid moths.. *Journal of Biophysical and Biochemical Cytology*.

[bibr89] Telfer W (1979). Sulfate and glucosamine labeling of the intercellular matrix in vitellogenic follicles in a moth.. *Roux's Archives of Developmental Biology*.

[bibr90] Telfer W, Om Adiyodi K, Adiyodi R (2002). Insect yolk proteins: a progress report.. *Reproductive Biology of Invertebrates*.

[bibr91] Telfer W, Rutberg L (1960). The effects of blood protein depletion on the growth of the oocytes in the Cecropia moth.. *Biological Bulletin*.

[bibr92] Telfer W, Melius M (1963). The mechanism of blood protein uptake by insect oocytes.. *American Zoologist*.

[bibr93] Telfer W, Anderson L (1968). Functional transformations accompanying the initiation of a terminal growth phase in the Cecropia moth oocyte.. *Developmental Biology*.

[bibr94] Telfer W, Smith D, Neville C (1970). Aspects of egg formation.. *Insect Ultrastructure. Symposia of the Royal Entomological Society*.

[birb95] Telfer W, Huebner E, Smith D, King R, Akai H (1982). The cell biology of vitellogenic follicles in *Hyalophora* and *Rhodnius*.. *Insect Ultrastructure*.

[bibr96] Telfer W, Keim P, Law J (1983). Arylphorin, a new protein from *Hyalophora cecropia*: comparisons with calliphorin and manducin.. *Insect Biochemistry*.

[bibr97] Telfer W, Pan M (1988). Adsorptive endocytosis of vitellogenin, lipophorin, and microvitellogenin during yolk formation in *Hyalophora*.. *Archives of Insect Biochemistry and Physiology*.

[bibr98] Telfer W, Pan M, Law J (1991). Lipophorin in developing adults of *Hyalophora cecropia*: support of yolk formation and preparation for flight.. *Insect Biochemistry*.

[bibr99] Tojo S, Betchaku T, Ziccardi V, Wyatt G (1978). Fat body protein granules and storage proteins in the silkmoth, *Hyalophora cecropia*.. *Journal of Cell Biology*.

[bibr100] VanAntwerpen R, Law J (1992). Lipophorin lipase from the yolk of *Manduca sexta* eggs: identificatioan and partial characterization.. *Archives of Insect Biochemistry and Physiology*.

[bibr101] Verhein A (1921). Die Eibildung der Museiden.. *Zoologische Jahrbuch, Abteilung für Anatomie und Ontogenie*.

[bibr102] Wang Y, Telfer W (1996). Cyclic nucleotide-induced termination of vitellogenin uptake by *Hyalophora cecropia* follicles.. *Insect Biochemistry and Molecular Biology*.

[bibr103] Wang Y, Telfer W (1997). Cyclic nuclotide-stimulated termination of vitellogenesis in *Hyalophora cecropia*: formation of a diffusion barrier and the loss of patency.. *Journal of Insect Physiology*.

[bibr104] Wang Y, Telfer W (1998). A. Cyclic AMP-induced water uptake in a moth ovary: inhibition by bafilomycin and anthracene-9-carboxylic acid.. *The Journal of Experimental Biology*.

[bibr105] Wang Y, Telfer W (1998). B. Pertussis toxin-sensitive G protein that supports vitellogenin uptake by promoting patency.. *Archives of Insect Biochemistry and Physiology*.

[bibr106] Wang Y, Telfer W (2000). Cyclic nucleotide-dependent protein phosphorylation in vitellogenic follicles of *Hyalophora cecropia*.. *Insect Biochemistry and Molecular Biology*.

[bibr107] Watson C, Sauman I, Berry S (1993). Actin as a major structural and functional element of the egg cortex of giant silkmoths during oogenesis.. *Developmental Biology*.

[bibr108] Wiemerslage L (1976). Lipid droplet formation during vitellognesis in the Cecropia moth.. *Journal of Insect Physiology*.

[bibr109] Williams C (1952). Physiology of insect diapause. IV. The brain and prothoracic glands as an endocrine system in the cecropia silkworm.. *Biological Bulletin*.

[bibr110] Woodruff R (1979). Electrotonic junctions in cecropia moth ovaries.. *Developmental Biology*.

[bibr111] Woodruff R, Telfer W (1990). Activation of a new physiological state at the onset of vitellogenesis in *Hyalophora* follicles.. *Developmental Biology*.

[bibr112] Woodruff R, Munz A, Telfer W (1992). Steady-state potentials in ovarian follicles of a moth, *Hyalopohora cecropia*.. *Journal of Insect Physiology*.

[bibr113] Woodruff R, Telfer W (1994). Steady-state gradient in calcium ion activity across the intercellular bridges connecting oocytes and nurse cells in *Hyalophora cecropia*.. *Archives of Insect Biochemistry and Physiology*.

[bibr114] Woodruff R, Dittmann F, Telfer W (1998). Ca^2+^ current from oocyte to nurse cells and suppression of uridine incorporation in the germinal vesicle of *Hyalophora cecropia*.. *Journal of Invertebrate Reproduction and Development*.

[bibr115] Wu Q, Brown M (2006). Signaling and functions of insulin-like peptides in insects.. *Annual Review of Entomology*.

[bibr116] Wyatt G (1967). The biochemistry of sugars and polysaccharides in insects.. *Advances in Insect Physiology*.

[bibr117] Wyatt G, Davey K (1996). Cellular and molecular actions of juvenile hormone. II. Roles of juvenile hormone in adult insects.. *Advances in Insect Physiology*.

[bibr118] Yamashita O, Hasegawa K (1976). Diapause hormone in silkworm ovaries incubated in vitro; 14C-trehalose incorporation into glycogen.. *Journal of Insect Physiology*.

[bibr119] Yamashita O, Irae K (1980). Larval hatching from vitellogenin-deficient eggs developed in male hosts of the silkworm.. *Nature* (*London*).

[bibr120] Yamashita O, Hasegawa K, Kerkut G, Gilbert L (1985). Embryonic diapause.. *Comprehensive Insect Physiology, Biochemistry and Pharmacology*.

[bibr121] Yamauchi H, Yoshitake N (1984). Developmental stages of ovarian follicles of the silkworm, *Bombyx mori*.. *Journal of Morphology*.

[bibr122] Zeng F, Shu S, Park Y, Ramaswamy S (1997). Vitellogenin and egg production in the moth, *Heliothis virescens*.. *Archives of Insect Biochemistry and Physiology*.

[bibr123] Zhu J, Indrasith L, Yamashita O (1986). Characterization of vitellin, egg-specific protein, and 30 kDa protein from *Bombyx* eggs, and their fates during oogenesis and embryogenesis.. *Biochimica et Biophysica Acta*.

[bibr124] Zimowska G, Shirk P, Silhacek D, Shaaya W (1995). A. Vitellin and formation of yolk spheres in vitellogenic follicles of the moth, *Plodia interpunctella*.. *Archives of Insect Biochemistry and Physiology*.

[bibr125] Zimowska G, Shirk P, Silhacek D, Shaaya W (1995). B. Termination of vitellogenesis in follcles of the moth, *Plodia interpunctella*: changes in oocyte and follicular epithelial cell activities.. *Archives of Insect Biochemistry and Physiology*.

